# Elevated High-Sensitivity Troponin and NT-proBNP Values in Febrile Children

**DOI:** 10.1097/PEC.0000000000003097

**Published:** 2023-12-20

**Authors:** Dorine M. Borensztajn, Chantal D. Tan, Yolanda de Rijke, Nienke N. Hagedoorn, Sascha C. Verbruggen, Henriette A. Moll, Clementien L. Vermont

**Affiliations:** From the ∗ Department of General Pediatrics, Erasmus MC, Sophia Children's Hospital, Rotterdam, the Netherlands; † Department of pediatrics, Northwest Clinics, Alkmaar, the Netherlands; ‡ Department of Clinical Chemistry, Erasmus MC, University Medical Center Rotterdam, Rotterdam, the Netherlands; § Intensive Care Unit, Department of Pediatrics and Pediatric Surgery, Erasmus MC - Sophia Children's Hospital, Rotterdam, Netherlands; ∥ Department of Pediatric Infectious Diseases & Immunology, Erasmus MC Sophia Children's Hospital, Rotterdam, the Netherlands.

**Keywords:** multisystem inflammatory syndrome in children, fever, N-terminal probrain natriuretic peptide, high-sensitivity troponin

## Abstract

**Objectives:**

The COVID-19 pandemic and subsequent rise of multisystem inflammatory syndrome in children have raised interest in high-sensitivity troponin (hs-TnT) and N-terminal probrain natriuretic peptide (NT-proBNP) because these have been found to be elevated in many cases of multisystem inflammatory syndrome in children. Our aim was to study hs-TnT and NT-proBNP concentrations in febrile children not affected by COVID-19.

**Methods:**

We retrospectively measured cardiac markers, hs-TnT, and NT-proBNP in leftover blood samples of febrile children (0–18 years) diagnosed and treated in a single-center emergency department (ED) (N = 67) and pediatric intensive care unit (PICU) (N = 19) that participated in a multicenter, prospective study of infection biomarkers (PERFORM).

**Results:**

Concentrations of hs-TnT, median 1.8 ng/L (interquartile range [IQR], 0.0–15.1), and NT-proBNP, 194 pg/mL (IQR, 54.9–706), were higher in febrile children than in controls (N = 25, hs-TnT 0.0 [IQR, 0–0]; NT-proBNP 56.3 [IQR, 29.7–109], both *P* < 0.001), whereas PICU patients had higher concentrations (hs-TnT 15.1 [IQR, 10.3–102] and NT-proBNP 828 [IQR, 657–4712], both *P* < 0.001) than ED patients (hs-TnT 0 [IQR, 0–7.4] and NT-proBNP 104 [IQR, 39.5–363]). No differences were found between viral and bacterial infections. Highest concentrations were found in children with either comorbidity predisposing to elevated concentrations (eg, chronic cardiac or renal disease) or children with critical illness or multiorgan failure such as those with septic shock.

**Conclusions:**

Concentrations of hs-TnT and NT-proBNP are often elevated in febrile children with different causes of fever. Concentrations were higher in children admitted to the PICU than in children attending the ED, and seem to reflect disease severity rather than the underlying cause of fever.

The COVID-19 pandemic and subsequent cases of multisystem inflammatory syndrome in children (MIS-C) have raised interest in the use of high-sensitivity troponin (hs-TnT) and N-terminal probrain natriuretic peptide (NT-proBNP) as diagnostic markers because these have been found to be elevated in many MIS-C cases as a sign of cardiac involvement^1^ and are currently used either as part of the definition or as an additional diagnostic criterion by the World Health Organization and the Royal College of Pediatrics and Child Health.^1^

Pre-proBNP is produced in response to increased myocardial stretch and wall stress, which is then cleaved into biologically active BNP and the inactive byproduct NT-proBNP. Elevated NT-proBNP concentrations can be found in many circumstances involving primary cardiac dysfunction, as well as secondary cardiac involvement due to pulmonary disease, sepsis, renal failure, liver cirrhosis, or intracranial pathologies.^2–5^ Troponins are cardiac regulatory proteins; their elevation indicates the presence of myocardial injury. In addition to acute cardiac pathology such as myocardial infarction, myocarditis, and heart failure, similar to NT-proBNP, troponins can be elevated in conditions such as pulmonary embolism, sepsis, critical illness, or renal disease.^6^

These studies suggest that, although primarily known as cardiac markers, hs-TnT and NT-proBNP can be elevated in secondary cardiac involvement or noncardiac disease as well, and therefore possibly could be interpreted as a marker of multiorgan involvement or disease severity in general and not solely as a specific diagnostic marker for cardiac disease.

Our aim was to study hs-TnT and NT-proBNP concentrations in children presenting to the emergency department (ED) or admitted to the pediatric intensive care unit (PICU) in the pre-COVID era with various causes of fever.

## MATERIALS AND METHODS

### Study Design

This study is a substudy of the Biomarker Validation Study (BIVA), which is embedded in the PERFORM project: Personalized Risk assessment in Febrile illness to Optimize Real-life Management across the European Union, a multicenter European trial. This study aims to develop and validate new tools to distinguish bacterial from viral infections. In the BIVA study, patients were prospectively recruited at the ED (BIVA-ED) or PICU (BIVA-PICU), and patient blood samples were collected; clinical data were extracted from electronic patient records. Ethical approval was obtained by the medical ethical committees (Commissie Mensgebonden Onderzoek, ID:NL58103.091.16) of the Radboud Universitair Medisch Centrum and Erasmus MC-Sophia Children's Hospital (EMC) and written informed consent from both parents and children aged 12 years or older was taken before sample collection. All children received follow-up by clinical visit or by a telephone interview at 48 to 72 hours and 28 days postinclusion to document any reattendances of readmission. For this substudy, only children from the EMC were included.

### Study Population and Setting

The study was conducted from 2017 until 2019 in the EMC Sophia, a large university medical center in the Netherlands. Children aged 0 to 18 years presenting with fever, a history of fever in the last 3 days, or suspected infection, who were considered ill enough to warrant blood tests, were included in the BIVA study. In this substudy, we included 3 groups of children: 1) children who fulfilled these inclusion criteria who attended the ED and were either admitted to the general ward or discharged home, 2) children admitted to the PICU either through the ED or after transfer from another hospital with a suspected community-acquired infection, and 3) afebrile preoperative healthy children as controls without major comorbidity who underwent minor surgery.

### Data Collection and Definitions

Clinical data were collected as part of routine clinical care using electronic patient health records. Data collected included general characteristics such as comorbidity and clinical information such as vital signs, diagnostic tests, and treatment.

Patients with chronic cardiac disease, chronic pulmonary disease, or chronic renal disease were considered to be predisposed to having chronically elevated cardiac markers.^2^

Vital signs were classified as abnormal according to Advanced Pediatric Life Support age-based reference ranges.^7^

The presumed cause of infection was determined by the research team based on routine data recorded in the patient's health record, using a previously published flowchart (Figure Supplemental Digital Content 1, http://links.lww.com/PEC/B175).^8^

In addition to the focus of the fever (eg, respiratory tract infection), this flowchart was used to classify the presumed cause of infection for each visit into “presumed bacterial,” “presumed viral,” “unknown bacterial or viral,” or “other” depending on clinical signs, C-reactive protein, and microbiological tests (bacterial cultures, viral, or bacterial polymerase chain reaction).

Reduced kidney function was defined as an increase in creatinine of more than 50% (stage 1) or more than 100% (stage 2 or 3)^9^ in comparison to normal concentrations for that patient when available or normal upper limit for age if not available or chronically elevated in a particular patient.

For this substudy, we retrospectively measured cardiac markers in leftover samples from the original BIVA study. The samples consisted of K_3_-EDTA plasma stored at −80°C. Cardiac markers included hs-TnT and NT-proBNP and were measured in the department of Clinical Chemistry by an electrochemiluminescence immunoassay on a cobas e 601 immunoassay analyzer (Roche Diagnostics, Almere, the Netherlands).

### Data Analysis

Descriptive statistics were used for general and clinical characteristics of our study population stratified for controls, children attending the ED, and children admitted to the PICU. Concentrations of hs-TNT and NT-proBNP were described in median and interquartile range (IQR). We used χ^2^ test for categorical variables and Mann-Whitney *U* test for numerical variables. A *P* value of less than 0.05 was determined as statistically significant. In addition, we described hs-TNT and NT-proBNP concentrations in the ED and PICU population stratified for viral and bacterial causes of infection. Furthermore, we compared children with a low ED-PEWS versus a high Emergency Department Pediatric Early Warning Score,^10^ a low lqSOFA versus a high Liverpool quick Sequential Organ Failure Assessment Score (LqSOFA)^11^ and less than 2 abnormal vital signs versus 2 or more abnormal vital signs.

Because several types of comorbidities can lead to chronic elevation of cardiac markers, [Eerola 2010; Sugimoto 2015;^2,12,13^ we performed an additional sensitivity analysis excluding children with any congenital heart disease, chronic pulmonary disease (cystic fibrosis, bronchopulmonary dysplasia), and chronic renal disease with reduced kidney function.^2^ Lastly, we described the characteristics of the children with the 10% highest concentrations of hs-TnT and/or NT-proBNP. A cut-off 10% was chosen arbitrarily to provide some more in-depth clinical details of these patients.

## RESULTS

### Concentrations of Hs-TnT and NT-proBNP in Febrile Children Versus Controls

We analyzed the blood samples of 111 children of which 61% were boys. Age ranged between 2 months and 17 years (Table 1). Of 111 children, 67 presented to the ED, 19 were admitted to the PICU, and 25 were preoperative controls. No significant differences were found in basic patient characteristics (age, sex, comorbidity) between ED patients and PICU patients.

**TABLE 1:** Patient Characteristics

	ED N = 67 N (%)	PICU N = 19 N (%)	Viral N = 26 N (%)	Bacterial N = 49 N (%)	Unknown N = 11 N (%)	Controls N = 25 N (%)
Age in years, median (IQR)*	9.8 (4.5–14.0)	8.4 (1.3–14.7)	7.5 (3.5–11.8)	9.8 (3.3–14.7)	12.5 (10.9–16.1)	9.0 (3.3–13.2)
Male*	41 (61)	12 (63)	17 (65)	32 (65)	4 (36)	15 (60)
Relevant comorbidity^†*^	13 (19)	4 (21)	10 (39)	7 (14)	0 (0)	0 (0)
Triage urgency (ED patients only)						
High: immediate, very urgent, intermediate	58 (87)	na	17 (85)	32 (89)	9 (81)	
Low: non-urgent, standard	9 (13)	na	3 (15)	4 (11)	2 (18)	
Ill appearance (ED patients only)	20 (30)	na	5 (25)	14 (39)	1 (9)	
Vital signs						
Tachypnea^‡^	12 (18)	10 (53)	5 (19)	16 (33)	1 (9)	
Hypoxia, oxygen saturation <94%	0 (0)	3 (16)	0 (0)	3 (6)	0 (0)	
Oxygen therapy	4 (6)	16 (84)	7 (27)	13 (27)	0 (0)	
Tachycardia^‡^	27 (40)	8 (42)	10 (39)	23 (47)	2 (18)	
Hypotension^‡^	2 (3)	6 (32)	2 (8)	6 (12)	0 (0)	
Prolonged capillary refill ≥3	2 (3)	10 (53)	3 (12)	9 (18)	0 (0)	
Reduced consciousness	2 (3)	10 (53)	5 (19)	7 (14)	0 (0)	
PICU severity scores						
PELOD-2 score, median (IQR)	na	2.0 (1–13)	na	na	na	
PRISM score, mean (IQR)	na	14.0 (8–19.0)	na	na	na	
Kidney function						
Normal	54 (81)	11 (58)	20 (77)	35 (71)	10 (91)	
Stage 1 renal failure	2 (3)	4 (21)	3 (12)	3 (6)	0 (0)	
Stage 2 or 3 renal failure	4 (6)	4 (21)	1 (4)	7 (14)	0 (0)	
Focus of infection						
Respiratory	17 (25)	8 (42)	15 (58)	10 (20)	0 (0)	
Gastrointestinal	6 (9)	1 (5)	2 (8)	5 (10)	0 (0)	
Urinary tract	10 (15)	0 (0)	0 (0)	10 (20)	0 (0)	
Skin, soft tissue, musculoskeletal	5 (8)	1 (5)	0 (0)	5 (10)	1 (9)	
Sepsis/meningitis	11 (16)	6 (32)	1 (0)	16 (33)	0 (0)	
Fever without focus	9 (13)	3 (16)	8 (31)	3 (6)	1 (9)	
Inflammatory	9 (13)	0 (0)	0 (0)	0 (0)	9 (82)	
Hospital admission in days, median (IQR)	4.5 (3.3–7.0)					
ICU admission in days, median (IQR)		6.0 (3.0–20.0)				
Death in this disease episode	0 (0)	1 (5)	0 (0)	1 (2)	0 (0)	

Missing values: general patient characteristics (age, sex, comorbidity, triage urgency): none. Ill appearance 6%. Vital signs: respiratory rate 38%; oxygen saturation 7%; oxygen therapy 6%; heart rate 9%; blood pressure 26%; capillary refill 37%; level of consciousness 5%. PELOD-2/PRISM: none. Kidney function 8%. Focus of infection none.

*No significant differences between ED and PICU patients.

^†^Patients with a history predisposing them to elevated cardiac enzymes: chronic cardiac disease, chronic pulmonary disease, or chronic renal disease.^2^

^‡^According to Advanced Pediatric Life Support reference values for age.

**FIGURE 1:**
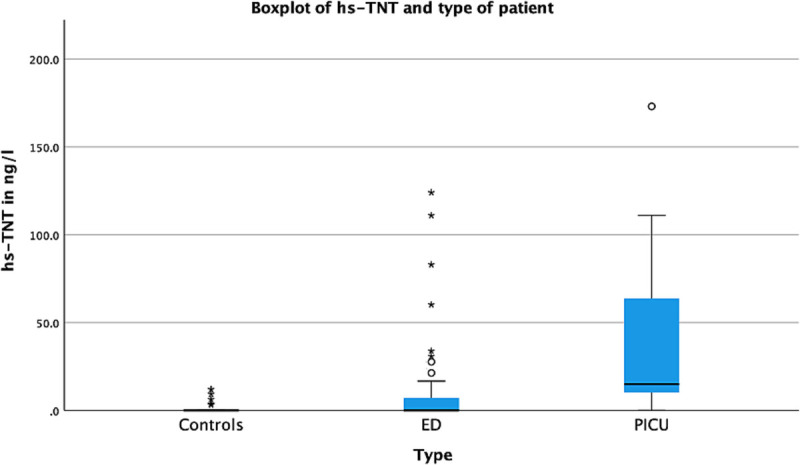
Concentrations of hs-TnT for different patient groups. Most extreme concentration removed from boxplot: 1169 ng/L.

**FIGURE 2:**
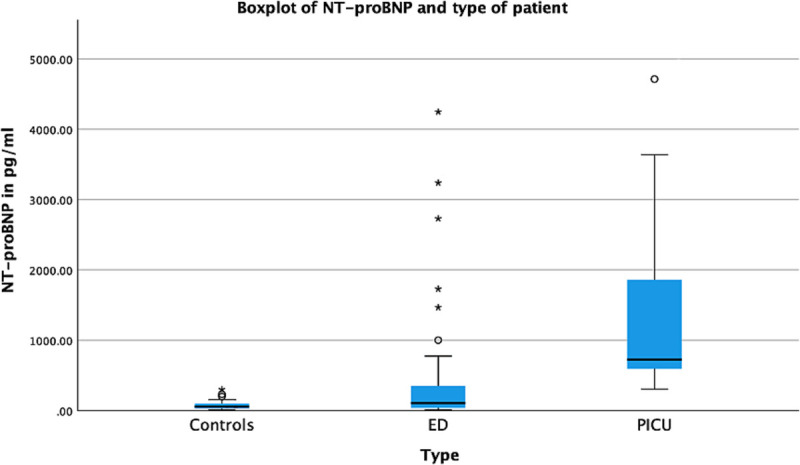
Concentrations of NT-pro-BNP for different patient groups. Most extreme concentrations removed from boxplot: 13,974, 14,542, 24,322, and 25,016 pg/mL.

Median hs-TnT and NT-proBNP concentrations were higher in the group of febrile children (hs-TnT 1.8 ng/L [IQR, 0.0–15.1]; NT-proBNP 194 pg/mL [IQR, 54.9–706]) than in healthy controls (hs-TnT 0.0 ng/L [IQR, 0.0–0.0]; NT-proBNP 56.3 pg/mL [IQR, 29.7–109], Fig. 1 and 2, Table 2).

**TABLE 2:** Concentrations of Hs-TnT and NT-Pro-BNP for Different Patient Groups

	Hs-TnT (Median, IQR)	*P*	NT-Pro-BNP (Median, IQR)	*P*
Overall		<0.01		<0.001
Controls (N = 25)	0 (0–0)		56.3 (29.7–109)	
Febrile children (N = 86)	1.8 (0–15.1)		194 (54.9–706)	
Setting		<0.01		<0.001
ED (N = 67)	0 (0–7.4)		104 (39.5–363)	
PICU (N = 19)	15.1 (10.3–102)		828 (657–4712)	
ED disposition		<0.05		<0.001
Discharged home from the ED (N = 29)	0 (0–0)		36.6 (23.3–180)	
Admitted to the general ward from the ED (N = 38)	3.6 (0–10.1)		190 (91.7–402)	
Type of infection		NS		NS
Viral (N = 26)	7.9 (0–15.2)		198 (62.5–730)	
Bacterial (N = 49)	3.8 (0–19.7)		331 (81.6–776)	
Patients with history predisposing to elevated cardiac enzymes excluded*		<0.05		<0.01
Controls (N = 25)	0 (0–0)		56.3 (29.7–109)	
Febrile children (N = 69)	0 (0–9.5)		131 (39.6–415)	
Vital signs^†^		<0.001		<0.001
<2 Abnormal vital signs (N = 61)	0 (0–9.9)		114 (38.1–371)	
>2 Abnormal vital signs (N = 25)	10.3 (5.6–24.9)		657 (317–2962)	

*Patients with a previous history of chronic cardiac disease, chronic pulmonary disease, or chronic renal disease (N = 17) (*Tsai S-H, Lin Y-Y, Chu S-J, Hsu C-W, Cheng S-M. Interpretation and use of natriuretic peptides in non-congestive heart failure settings. Yonsei Med J. 2010;51:151–163*).

^†^Heart rate, oxygen saturation, respiratory rate, blood pressure, capillary refill, and level of consciousness. Vital signs were classified as abnormal according to Advanced Pediatric Life Support age-based reference ranges (*Turner N. Advanced pediatric life support. Bohn Stafleu van Loghum; 2017*).

Results remained significant after excluding children with relevant comorbidity (n = 17, with 69 febrile children and 25 controls remaining for the analysis, Table 2).

Concentrations of hs-TnT and NT-proBNP were significantly higher in children with 2 or more abnormal vital signs (Table 2) in comparison with children with no abnormal vital signs or 1 abnormal vital sign.

Details regarding the 16 patients with the 10% highest concentrations of hs-TnT and/or NT-proBNP are displayed in Supplemental Digital Content 2, http://links.lww.com/PEC/B176. Ten of these patients were admitted to the PICU, 4 were seen at the ED and admitted to the general ward, and 2 were seen at the ED and discharged home. All patients displayed in Supplemental Digital Content 2, http://links.lww.com/PEC/B176, had either signs of multiorgan failure/critical illness (n = 8), comorbidity predisposing them to elevated cardiac markers (n = 6), or both (n = 2).

### Concentrations of Hs-TnT and NT-proBNP in Febrile Children at the ED Versus Febrile Children Admitted to the PICU

Concentrations of hs-TnT were higher for PICU patients (15.1 ng/L [IQR, 10.3–102]) than for ED patients (0.0 ng/L [IQR, 0.0–7.4]). Similarly, NT-proBNP concentrations were higher for PICU patients (828 pg/mL [IQR, 657–4712]) than for ED patients (104 pg/mL [IQR, 39.5–363], *P* < 0.001, Fig. 1 and 2, Table 2). In addition to this, concentrations of hs-TnT and NT-proBNP were significantly higher in patients admitted to the general ward from the ED than in patients discharged home from the ED (Table 2).

### Concentrations of Hs-TnT and NT-proBNP in Febrile Children With Viral Disease Versus Bacterial Disease

Of the 86 ED and PICU patients, 49 (57%) were classified as having a definite or probable bacterial infection, 26 (30%) were classified as definite or probable viral infections, and 11 (13%) were classified as other causes of fever (Table 1).

No significant differences were found in the concentrations of hs-TnT or NT-proBNP in children classified as viral versus bacterial disease (Table 2).

## DISCUSSION

### Main Findings

Our data show that hs-TnT and NT-proBNP are frequently elevated in febrile children presenting to the ED or admitted to the PICU in comparison with healthy controls. Concentrations of hs-TnT and NT-proBNP were significantly higher in patients admitted to the PICU than ED patients admitted to the general ward or discharged home.

No differences were found between children with viral disease versus bacterial disease, which suggests that hs-TnT and NT-proBNP concentrations are related to disease severity more than to the underlying cause of infection, although our sample might have been too small to detect differences between these groups.

### Findings in Relation to Previous Literature

The use of different test variants (eg, hs-TnT T, hs-TnT I, hs-TnT, and BNP versus NT-proBNP), different assays, and different units makes comparison between studies less straightforward.^14,15^ Despite this, it is still possible to compare overall trends.

Similar to previous studies, we found elevated concentrations of hs-TnT and NT-proBNP in children with sepsis admitted to the PICU and children with bronchiolitis.^5,16,17^ However, we also found elevated concentrations in children with bacterial and viral infections not admitted to the PICU.

The finding that PICU patients had higher concentrations than ED patients is in line with previous studies that found the level of cardiac markers to be associated with sepsis severity and mortality in pediatric patients.^18,19^

Our data highlight how cardiac markers can be elevated in febrile children with fever of different origins due to several reasons and are not specific for a certain diagnosis. For example, several viral illnesses have been linked to a variety of cardiac manifestations, which are often self-limiting.^20^ As previously described, we found especially high concentrations in critically ill children such as those with multiorgan failure, septic shock, and renal failure, or in children with a history predisposing them to elevated cardiac markers, such as congenital heart disease or chronic pulmonary disease. Although the median concentrations of hs-TnT and NT-proBNP were higher in a cohort of children with MIS-C than in our study, there was considerable overlap because some of our patients had concentrations of approximately 25,000 pg/mL.^21,22^

The NT-proBNP concentrations of healthy controls were higher than those usually found in healthy adults, as has been described before by Nir et al.^15^ However, except 1, all were within the previously described reference ranges in healthy children.^15^

Although previous studies have described elevations of hs-TnT and NT-proBNP in children with sepsis and a number of other diseases, to our knowledge, our study is the first study that describes these elevations in a heterogeneous population of febrile children, including non-critically ill children attending the ED with fever. These findings should ensure that clinicians in the ED maintain a broad perspective when assessing patients.

### Implications for Clinical Practice and Research

Elevations of hs-TnT and NT-proBNP are currently used to support the diagnosis of MIS-C.^1^ However, our data show that hs-TnT and NT-proBNP can be elevated in a broad spectrum of febrile illnesses, such as sepsis, critical illness, multiorgan involvement, or chronic comorbidity. Because MIS-C can have considerable clinical overlap with other illnesses such as sepsis or meningitis,^23^ and no sign or symptom is pathognomonic for MIS-C,^24^ more research is needed to evaluate whether more specific diagnostic criteria can be established to help differentiate between MIS-C and other causes of fever in sick children. Future studies should include children with MIS-C as well as children with a different diagnosis to be able to make a direct comparison between these groups and establish accurate cut-off concentrations.

### Strengths and Limitations

To our knowledge, this is the first study describing hs-TnT and NT-proBNP concentrations in a diverse population of febrile children regarding disease severity and the underlying cause. We collected extensive clinical details including details on comorbidity that could predispose children to having chronically elevated cardiac markers.

Although we only studied a small sample, and therefore might not have captured the full range of laboratory abnormalities and true differences between study groups, our sample did show significant elevation of hs-TnT and NT-proBNP concentrations in febrile children without MIS-C. Another limitation is that we did not include children with MIS-C in our study, precluding a direct comparison with this patient group. However, by studying children who presented in the pre-COVID era, we can be sure that the elevations were not attributable to MIS-C.

A further limitation is that in children with comorbidity, preillness concentrations were not performed, and therefore it is unclear if the elevation can be attributed to the preexisting illness, the intercurrent febrile episode, or both. However, differences between groups remained significant after excluding children with relevant comorbidity. Studying a larger sample with febrile children with and without comorbidity could further clarify this. We deliberately did not exclude children with comorbidity because these form a substantial part of febrile children attending the ED and therefore should not be left out of scientific studies.^25^

Another limitation is that cardiac tests were not routinely performed and thus were only performed in a minority of patients. This is due to the retrospective nature of the study and perhaps due to the low index of suspicion of severe or primary cardiac disease in many (ED) patients.

*Due to this limitation, we were unable to determine whether the elevation of cardiac markers was due to cardiac dysfunction resulting from primary cardiac disease or secondary cardiac involvement due to sepsis or other factors such as pulmonary or renal disease.*

*However, several previous studies showed a wide variety of acute as well as chronic disease to increase these markers, including several illnesses without cardiac involvement.* ^2–6^

*Furthermore, it is unlikely that most children attending the emergency department (ED) and subsequently discharged from either the ED or the general ward, had severe (primary) cardiac dysfunction.*

*In future research, it would be beneficial to include cardiac tests to further investigate the presence or absence of cardiac injury and/or dysfunction and its exact causes in febrile children attending the ED.*

The final limitation is that due to the use of different assays, comparison of hs-TnT and NT-proBNP concentrations between studies should be performed with caution; however, the validity of our results is supported by the fact that in our study, we used the same assay for febrile children and healthy controls.

## CONCLUSIONS

Febrile children presenting to the ED or admitted to the PICU with viral as well as bacterial disease can have significant elevations of hs-TnT and NT-proBNP concentrations. In our study, hs-TnT and NT-proBNP concentrations were higher in children admitted to the PICU than in children admitted to the general ward or discharged from the ED. Moreover, the concentrations were found to be higher in children with more abnormal vital signs, providing supporting evidence that these heightened levels are reflective of multiorgan involvement and disease severity rather than a specific diagnosis.
